# Platelet-Rich Plasma Promotes the Proliferation of Human Muscle Derived Progenitor Cells and Maintains Their Stemness

**DOI:** 10.1371/journal.pone.0064923

**Published:** 2013-06-07

**Authors:** Hongshuai Li, Arvydas Usas, Minakshi Poddar, Chien-Wen Chen, Seth Thompson, Bahar Ahani, James Cummins, Mitra Lavasani, Johnny Huard

**Affiliations:** Department of Orthopedic Surgery, Stem Cell Research Center, University of Pittsburgh, Pittsburgh, Pennsylvania, United States of America; Clinica Universidad de Navarra, Spain

## Abstract

Human muscle-derived progenitor cells (hMDPCs) offer great promise for muscle cell-based regenerative medicine; however, prolonged *ex-vivo* expansion using animal sera is necessary to acquire sufficient cells for transplantation. Due to the risks associated with the use of animal sera, the development of a strategy for the ex vivo expansion of hMDPCs is required. The purpose of this study was to investigate the efficacy of using platelet-rich plasma (PRP) for the *ex-vivo* expansion of hMDPCs. Pre-plated MDPCs, myoendothelial cells, and pericytes are three populations of hMDPCs that we isolated by the modified pre-plate technique and Fluorescence Activated Cell Sorting (FACS), respectively. Pooled allogeneic human PRP was obtained from a local blood bank, and the effect that thrombin-activated PRP-releasate supplemented media had on the *ex-vivo* expansion of the hMDPCs was tested against FBS supplemented media, both *in vitro* and *in vivo*. PRP significantly enhanced short and long-term cell proliferation, with or without FBS supplementation. Antibody-neutralization of PDGF significantly blocked the mitogenic/proliferative effects that PRP had on the hMDPCs. A more stable and sustained expression of markers associated with stemness, and a decreased expression of lineage specific markers was observed in the PRP-expanded cells when compared with the FBS-expanded cells. The *in vitro* osteogenic, chondrogenic, and myogenic differentiation capacities of the hMDPCs were not altered when expanded in media supplemented with PRP. All populations of hMDPCs that were expanded in PRP supplemented media retained their ability to regenerate myofibers *in vivo*. Our data demonstrated that PRP promoted the proliferation and maintained the multi-differentiation capacities of the hMDPCs during *ex-vivo* expansion by maintaining the cells in an undifferentiated state. Moreover, PDGF appears to be a key contributing factor to the beneficial effect that PRP has on the proliferation of hMDPCs.

## Introduction

Skeletal muscle is a good source of various cellular progenitors with potential musculoskeletal therapeutic applications [1], [2], [3]. A population of cells has been isolated by a modified pre-plate technique from mouse skeletal muscle, that when compared to myoblasts, display a superior regeneration capacity in various musculoskeletal tissues, including skeletal and cardiac muscles, bone, and articular cartilage [4], [5], [6], [7]. When compared to myoblasts, these cells, termed *muscle-derived stem cells* (MDSCs) [8], demonstrated the capacity for self-renewal, long term proliferation, multi-potent differentiation, and a superior ability to survive, due to their increased resistance to oxidative and inflammatory stresses [9]. Several populations of human muscle-derived progenitor cells, including satellite cells [10], [11], myo-endothelial cells [12], and pericytes [2], [3], [13], [14], [15], [16] have also been isolated using the pre-plate technique and Fluorescence Activated Cell Sorting (FACS), respectively [12], [16]. These muscle-derived cells are multi-potent progenitor cells that exhibit similar multi-lineage differentiation potentials and can differentiate into muscle, bone, cartilage, and fat both *in vitro* and *in vivo* [12], [16]. In the current study we refer to these different populations of cells collectively as human muscle-derived progenitor cells (hMDPCs). hMDPCs offer great promise for muscle cell-based regenerative medicine; however, due to their relative scarcity (MDSCs: 0.05%–0.1%; Myoendothelial cells: 0.4±0.1% [12]; Pericytes: 0.29±0.09% [16]), prolonged *ex-vivo* expansion is necessary to acquire sufficient cell numbers for therapeutic transplantation. This involves exposing the stem cells to commercial animal sera such as fetal bovine serum (FBS) or fetal calf serum (FCS), and/or to growth factors and other supplements such as chicken embryo extract (CEE). Due to the risks associated with the use of these animal sera [17], [18], the development of an appropriate strategy for hMDPCs *ex-vivo* expansion is required.

Platelet-rich plasma (PRP) can be rapidly and easily obtained by centrifugal separation from whole blood. Multiple growth factors are concentrated in PRP at high levels after centrifugation, hence, PRP obtained from patients can be used as an autologous source of growth factors for various tissue repairs [19], [20], [21], [22], [23]. The introduction of PRP into clinical practice was originally suggested by Marx *et al*. [24] in 1998. In addition to being autologous in nature, and therefore posing no risk of disease transmission or immunogenic reaction, PRP offers the advantage of being a growth factor concentrate containing the “right” factors at the “right” proportions necessary for aiding the healing process of various tissues. Despite the expanding potentials of directly using PRP for accelerating the healing of a variety of tissues, there is also a rising interest in combining PRP with mesenchymal stem cells (MSCs) for tissue regeneration applications. PRP is a promising supplement for *ex-vivo* cell expansion [25], [26] or as a PRP-gel delivery vehicle for cells during transplantation [27], [28]. Several studies have suggested that PRP could be used as a supplement for *ex-vivo* expansion of mesenchymal stem cells from bone marrow [25], [29], [30] and adipose tissues [31]; however, no studies have been conducted on the effects PRP has on muscle derived progenitor cells. In the current study, we hypothesized that PRP could be a promising candidate for the *ex vivo* expansion of hMDPCs and we investigated the effect that PRP had on the proliferation and multi-lineage differentiation capacities of the hMDPCs *in vitro*. We also investigated the efficiency of PRP-expanded hMDPCs for regenerating injured muscle *in vivo*.

## Materials and Methods

### Isolation and Cultivation of hMDPCs

Three populations of hMDPCs were used in this study: Pre-plated muscle derived cells, myo-endothelial cells, and pericytes. All procedures were approved by the institutional review board at the University of Pittsburgh. Written informed consent was obtained from all subjects. Human pre-plated muscle derived cells (pre-plated MDPCs) were isolated by the pre-plate technique [32], [33], [34], [35] from three donors. Briefly, post-mortem skeletal muscle biopsies (1 to 2 g) were obtained from the National Disease Research Interchange (NDRI) from three male human subjects (50–70 years old) with no known musculoskeletal diseases. The tissue was finely minced and then serially digested with 0.2% collagenase, 0.25% dispase, and 0.1% trypsin, for one hour each. The digested tissue was then suspended in Dulbecco’s Modified Eagle’s Medium (DMEM, GIBCO) supplemented with 20% fetal bovine serum (FBS, GIBCO), 1% penicillin/streptomycin (PS, GIBCO), and mechanically dissociated by serially passaging the suspended tissue through 18-, 21-, and 23-gauge needles. The resultant cell suspension was then placed in a collagen-coated flask (0.1 g/L collagen type I, Sigma) (preplate (pp) 1). After one hour, non-adherent cells were transferred to a fresh collagen-coated flask (pp 2), and then 24 hours later, those in the second preplate were transferred to another fresh collagen-coated flask (pp 3). This procedure was repeated until pp6 was obtained. The pp6 cells were then expanded and used for this study. Myo-endothelial cells and pericytes were obtained from three donor muscle biopsies which were isolated via FACS as previously described [12], [16]. Briefly, fresh muscle tissue was cut into small pieces in DMEM containing 20% FBS, 1% PS, and then enzymatically dissociated with collagenases type I and II (1 mg/ml, Sigma). Cells were passed through a 70 µm cell strainer, centrifuged, and re-suspended in erythrocyte lysis buffer and incubated for 15 min at RT. The cells were then incubated with a combination of the following directly conjugated mouse anti-human antibodies: anti-CD34-PE (DAKO, 1∶100), anti-CD45-APC-Cy7 (Santa Cruz Biotechnologies, 1∶200), anti-CD56-PE-Cy7 (Serotec, 1∶100), anti-CD146-FITC (Serotec, 1∶100), and anti-CD144-PE (BECKMAN, 1∶200) for 15 min in the dark. Myo-endothelial cells (CD45^−^, CD56^+^, CD144^+^, and CD34^+^) and pericytes (CD45^−^, CD56^−^, CD146^+^, and CD34^−^) were sorted on a FACS-Aria flow cytometer (BD), and then cultured under standard conditions (see preparation of supplements and media). All hMDPCs were expanded and used between passages 5–8 for all the experiments.

### Preparation of Supplements and Media

For pre-plated MDPCs and myo-endothelial cells, DMEM-high glucose (4.5 g/l D-glucose) with L-glutamine and 110 mg/L sodium pyruvate (DMEM-HG), and 1% PS served as the basal media (BM). For pericytes, DMEM-HG without sodium pyruvate, and 1% PS served as the BM. Derivations on the BM used in the experimental methods are defined in the sections below, i.e. concentrations of FBS and PRP-releasate supplementation and specific media types for *in vitro* differentiation studies.

### Thrombin-activated PRP Releasate

Human PRP releasate was prepared according to a protocol previously described [29], [31] with some modifications. Six AB-blood-group-typed whole blood donations were used to prepare one pool of PRP derived from freshly prepared buffy coats (Central Blood Bank, Pittsburgh, PA, USA). All buffy coats were pooled together and centrifuged at 3000 g for 10 min at RT. After centrifugation, a platelet pellet formed at the bottom with the supernatant considered to be platelet poor plasma (PPP). At least half of the PPP was transferred to another tube; and the platelet pellet was then re-suspended in the remainder of the PPP to form the PRP. The concentration of the platelets within the PRP was determined using a hemocytometer, and standardized to 2×10^6^ platelets per microliter by adding a calculated amount of PPP. PRP was then activated with one unit per ml human thrombin (Sigma-Aldrich). After activation, the PRP releasate was separated from the cellular debris by centrifugation at 3000 g for 30 min, followed by filtration through a 0.2 µm filter. The PRP releasate was aliquoted and stored at −80 °C until usage.

### Enzyme-linked Immunosorbent Assay (ELISA)

The concentrations of transforming growth factor-beta1 (TGF-β1), platelet derived growth factor-AB (PDGF-AB), and vascular endothelial growth factor (VEGF) within the thrombin activated PRP releasate were measured using commercially available ELISA kits (DB100B, human TGF-beta1 immunoassay, Quantikine, R&D system; DHD00B, Human PDGF-AB immunoassay, Quantikine, R&D system; DVE00, human VEGF immunoassay, Quantikine, R&D system).

### Proliferation Assays

A DNA assay was used to determine cell growth. Briefly, 2×10^3^ hMDPCs were seeded on a 48 well plate in BM and incubated overnight. The next day, depending on the different experimental designs, the media was changed to different conditioned media. At days one, three, and five, after the initial media change, the cell lysates were prepared by the addition of 200 µl 0.1% Triton X-100 (Sigma-Aldrich), followed by three freeze-thaw cycles. A 50 µl aliquot of the cell lysate was used to determine double-stranded DNA (dsDNA) content, which was measured using a Quant-iT dsDNA high-sensitivity assay kit (Invitrogen, USA).

Long-term proliferation kinetics were tested as described previously [29], [36]. Cells were counted and passaged at a confluence of 70–80% up to eight passages. At each passage, the population doubling (PD) rate was determined using the formula x = [log10(Nh)-log10(N1)]/log10(2), where N1 is the plated cell number and Nh is the cell number at harvest. The PD of each passage was calculated and added to the PD of the previous passages to generate the cumulative population doubling rate (CPD).

### Neutralization Assay

2×10^3^ hMDPCs were seeded onto a 48 well plate in BM. Twenty-four hours later, the media were changed to different neutralization-antibody-conditioned media, which were prepared by adding the following neutralizing antibodies (Abs) to the culture media supplemented with 10% PRP, and gently agitated at 4°C for 1 h: neutralization Abs against VEGF (AF-293-NA, neutralizing the biological activity of VEGF_165_ and VEGF_121_; R&D System), PDGF (AB-20-NA, neutralizing the biological activity of natural human PDGF including PDGF-AB, BB, & AA; R&D Systems), or TGF-β1 (TB21; neutralizing the biological activity of human TGF-β1; Abcam); and low endotoxin isotype control Abs (0109-14, goat IgG, SouthernBiotech; 011-001-297, rabbit IgG, Rockland; 400124, mouse IgG, Biolegend) were also added as controls. The effects that the neutralized PRP had on the growth of the hMDPCs were determined using the DNA assay described in the Proliferation Assay section.

### Reverse-transcription PCR

Cellular RNA of hMDPCs was extracted using an RNeasy Mini Kit (Qiagen). Aliquots of 1 µg total RNA were hybridized with random primers and converted into cDNA using a SuperScript First-Stand Synthesis System (Invitrogen). The expressions of different gene markers were analyzed by semi-quantitative RT-PCR. RT-PCR products were analyzed on an agarose gel and visualized with ethidium bromide. Gel pictures were acquired using a Gel Doc 1000 (Biorad Laboratories, USA) and the densitometry analysis was performed with Molecular Analyst 2.1.2 (Biorad). The gene signals were normalized to β–actin. Oligonucleotide primers specific to the individual markers included in this study are presented in **Table 1**.

**Table 1:** List of primer sequences for the tested genes.

Genes	Accession No.	Forward	Reverse	b.p.
Nanog	NM-024865	CAGCCCCGATTCTTTCCACCAGTCCC	CGGAAGATTCCCAGTCGGGTTCACC	390
OCT4	NM_001159542	GTGTTCAGCCAAAAGACCATCT	GGCCTGCATGAGGGTTTCT	156
Sox-2	NM-003106	GGGAAATGGGAGGGGTGCAAAAGAGG	TTGCGTGAGTGTGGATGGGATTGGTG	150
CD105	U37439	AGCCCCACAAGTCTTGCAG	GCTAGTGGTATATGTCACCTCGC	84
CD73	NM_002526	GGCTCCTCTCAATCATGCCG	CCAGAACATTTCATCCGTGTGT	102
CD90	NM_006288	TCGCTCTCCTGCTAACAGTCT	CTCGTACTGGATGGGTGAACT	134
CD44	L05424	CCTGGGATTGGTTTTCATGGT	CCAGCCTGCTGAGATGGTATTT	107
PAX2	NM_153427	GCCGAGGACCCGTCTAAGA	TGCTGGCTGGTAAAGTGAGTC	62
BMPR-1A	NM_004329	TCAGACTCCGACCAGAAAAAGT	TGGCAAAGCAATGTCCATTAGTT	145
BMPR-1B	NM_001203	ATTTGCAGCACAGACGGATATT	GACACTGAAAATCTGAGCCTTCT	109
BMPR-2	NM_001204	CGGCTGCTTCGCAGAATCA	AGGTGCTACCTTTCGAGCATA	126
ALDH	NM_000689	GCACGCCAGACTTACCTGTC	CCTCCTCAGTTGCAGGATTAAAG	129
RUNX2	NM_004348	TCCTATGACCAGTCTTACCCCT	GGCTCTTCTTACTGAGAGTGGAA	190
ALP	NM_001127501	AAGGACGCTGGGAAATCTGTG	GTGGCATGGTTCACTCTCGT	56
SOX9	NM_000346	GCCAGGTGCTCAAAGGCTA	TCTCGTTCAGAAGTCTCCAGAG	213
Aggrecan	NM_013227	AGGAGACAGAGGGACACGTC	TCCACTGGTAGTCTTGGGCAT	249
CD56	NM_000615	ACATCACCTGCTACTTCCTGA	CTTGGACTCATCTTTCGAGAAGG	137
Desmin	NM_001927	AACCAGGAGTTTCTGACCACG	TTGAGCCGGTTCACTTCGG	137

### Flow Cytometry Analysis

Flow cytometry was performed on populations of hMDPCs that were expanded in FBS (20%) or PRP (20%) supplemented media for up to three weeks. The cells were first incubated with Abs against the appropriate cell surface markers for 30 min in the dark. The cells were then fixed with 0.4% paraformaldehyde, and permeabilized with 0.1% Triton X-100 for 30 min. After three washes with PBS, the cells were incubated with Abs against the appropriate intracellular markers for 30 min, and fixed again with 0.4% paraformaldehyde. The following mouse anti-human antibodies were used: CD146-FITC (Serotec), CD144-PE(BECKMAN), CD56-PE-Cy7(Serotec), CD34-APC (BD Pharmingen), CD45-APC-CY7 (BD Pharmingen), CD105-PE (Invitrogen), CD90-APC (BD Pharmingen), CD44-PE (Invitrogen), Nanog-Alexa Fluor 647 (BD Pharmingen), Oct3/4-PerCP-Cy5.5 (BD Pharmingen), Sox-2-V450 (BD Horizon), and HLA-DR-Brilliant Violet 570 (BioLegend). Labeled cells were acquired and analyzed using a FACScan flow cytometer running CellQuest software (Becton Dickinson). Comparative analysis was performed with FlowJo Version 7.6 (Tree Star, Inc., Ashland, OR, USA).

### *In vitro* Differentiation Assays

For osteogenic differentiation, micromasses of 2.5×10^5^ cells were formed by centrifugation at 400 g for 5 min and kept for 3 weeks in osteogenic media (DMEM containing 10% FBS, 100 nM dexamethasone, 10 mM β-glycerophosphate, and 0.05 mM L-ascorbic acid-2-phosphate). At weeks one, two, and three, cell pellets were examined with a MicroCT scanner (VivaCT 40, Scanco, Switzerland) and the mineralized bone volumes (BV) were documented and compared among the groups.

For chondrogenic differentiation, micromasses of 2.5×10^5^ cells were formed and kept for four weeks in DMEM, 100 nM dexamethasone, 0.05 mM L-ascorbic acid-2-phosphate, 1% ITS (insulin 25 µg/ml, transferrin 25 µg/ml, and sodium selenite 25 µg/ml), 0.35 nM proline, and 10 ng/ml recombinant human TGF-β3 (Lonza). Cell pellets were collected after four weeks, and histology and biochemical analyses were performed. For histology, randomly selected pellets (n = 2 per data point) were fixed in 4% paraformaldehyde, CMC (NEG50, Richard-Allan Scientific) embedded, and frozen in liquid nitrogen; 5 µm sections were cut using a cryostat (HM505E, MICROM, USA). Consecutive sections were stained with alcian blue. For biochemical analyses, randomly selected pellets (n = 3 per data point) were digested for 6 h at 60°C with 125 µg/ml papain in PBE buffer (100 mM phosphate, 10 mM EDTA, PH 6.5) containing 10 mM cysteine, by using 100 µl of enzyme per sample. Glycosaminoglycan (GAG) content was measured using dimethylmethylene blue dye and a spectrophotometer (Infinite M200, TECAN, NC, USA). Bovine chondroitin sulfate was used as a standard.

For myogenic differentiation, 2×10^4^ cells were seeded onto a 24 well plate and cultured for ten days in fusion media (BM with 2% FBS) which induces myogenic fusion of the cells into elongated, multinucleated myotubes. At the end of culture, the cells were fixed with cold methanol. After blocking with donkey serum, mouse anti-human-fast myosin heavy chain (f-MHC) (m4276, Sigma, 1∶250) was added and incubated overnight at 4°C. After washing with PBS, donkey-anti-mouse 594 (A21203, Invitrogen, 1∶500) was added for two hours at RT. The nuclei were labeled with 4′, 6-diamidino-2-phenylindole (DAPI). Five fields in each well were randomly chosen for analysis, and three replicate experiments were performed. The percentage of fast-MHC-expressing nuclei/total nuclei were documented and compared among groups.

### *In vivo* Transplantation of hMDPCs

Six to eight week old mdx-SCID mice (Jackson Laboratories, USA) were used in this study. All experiments were carried out in strict accordance with the recommendations in the Guide for the Care and Use of Laboratory Animals of the National Institutes of Health. The protocol was approved by the Animal Research and Care Committee of University of Pittsburgh. All surgery was performed under isoflurane anesthesia, and all efforts (subcutaneous injections of 1 mg/kg ketarolac, once daily for three days post-op) were made to minimize suffering. hMDPCs were expanded in PRP (20%) supplemented or FBS (20%) supplemented media for three weeks. 1×10^5^ cells were re-suspended in 20 µl PBS and transplanted intramuscularly in a single injection into the mdx-SCID’s gastrocnemius muscles that had been injured one day earlier by intramuscular injection of 20 µl cardiotoxin (0.15 µg/µl, CTX, Molecular Probes). Animals were sacrificed 28 days after injection and treated muscles were frozen as described previously. Human Major histocompatibility complex (MHC) class I staining was performed on acetone-fixed, 5% donkey serum-blocked sections using a rabbit anti-human MHC-class-I antibody (1∶600 dilution, ab52922, Abcam) and mouse anti-human mitochondria (1∶100 dilution, MAB1273, Millipore) to detect human cell-derived myofibers. Sections were then washed in PBS and incubated with an Alexa Fluor 594 labeled donkey-anti-rabbit IgG antibody (A21207, Invitrogen) and Alexa Fluor 488 donkey-anti-mouse IgG antibody (A21202, Invitrogen). Nuclei were stained with DAPI. Sections containing the highest number of fibers expressing human MHC-class-I positive myofibers were imaged using fluorescence photomicroscopy (Nikon Eclipse E800, Japan). Consecutive sections were stained with H&E, and the images were taken in the same area. The number of human MHC-class-I positive fibers per section were manually counted and reported as the number of hMHC-I positive fibers/1×10^5^ injected cells.

### Statistical Analysis

All experiments were repeated at least three times, and statistical tests were performed using SPSS 16.0 (SPSS, Inc., Chicago, IL, USA). Data were represented as the mean ± standard deviation (SD). Data were tested for normality and equal variance before analysis. Statistical differences were calculated using analysis of variance (ANOVA; or ANOVA on ranks if equal variance testing failed). Differences were considered significant at *P*<0.05.

## Results

### PRP Promotes the Proliferation of hMDPCs

The concentration of platelets in the pooled PRP was standardized to 2×10^6^ platelets per ml (see methods section). The concentrations of TGF-β1, PDGF-AB, and VEGF within the PRP releasate were 485.25±38.12 ng/ml, 297.4±41.26 ng/ml, and 648.73±79.45 pg/ml respectively.

**Figure 1:**
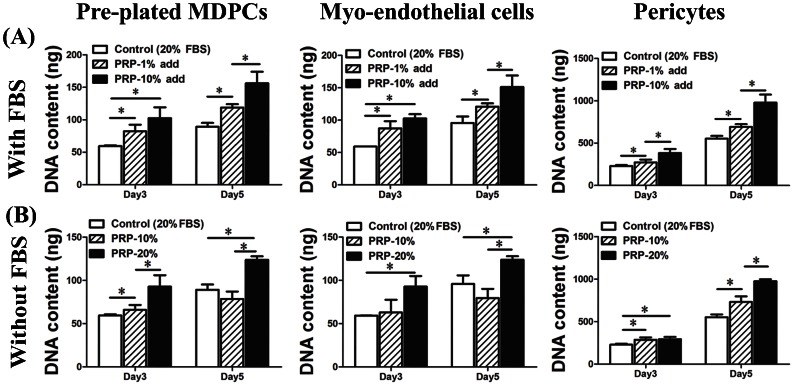
Influence of PRP on the proliferation of hMDPCs. **A**: Influence of PRP on the proliferation of hMDPCs in the presence of 20% FBS. n = 4, ^*^ *P*<0.05. Data showed that PRP can promote the proliferation of hMDPCs in a dose-dependent manner. **B**: Influence of PRP on the proliferation of hMDPCs in the absence of FBS. n = 4, ^*^ *P*<0.05. Data showed that PRP alone can promote the proliferation of hMDPCs in a dose-dependent manner, and 10% PRP induced comparable effect as the 20% FBS did on the proliferation of the hMDPCs.

The effect that PRP had on the proliferation of the hMDPCs in the presence of FBS was first tested. PRP (1% and 10% vol/vol) was added to the proliferation media (BM, with 20% FBS) inducing a significant increase in DNA content in all populations of hMDPCs in a dose-dependent manner from day three to day five compared to the control groups (**Fig. 1A**). In order to test if PRP alone without FBS would be sufficient to support hMDPCs proliferation, the DNA content of the hMDPCs cultured in the PRP-only supplemented media (BM with 10% or 20% PRP without FBS) was compared to the cells cultured with standard proliferation media (BM with 20% FBS). PRP alone induced proliferation of the hMDPCs in the absence of FBS in a dose dependent manner (**Fig. 1B**). 20% PRP significantly increased the proliferation of hMDPCs compared to the 10% PRP supplemented media. 10% PRP alone induced-proliferation at a comparable rate to that achieved with 20% FBS; a PRP concentration of 20% increased the proliferation rate of the cells by day five above that of using 20% FBS to 38.2%, 29.5%, and 76.1% in the pre-plated MDPCs, myo-endothelial cells, and pericytes, respectively.

**Figure 2:**
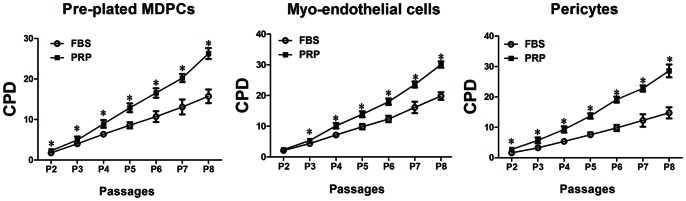
Influence of PRP on the long-term proliferation of hMDPCs. hMDPCs were cultured in 20% PRP (PRP group). The mean cumulative population doubling rate (CPD) was compared among the cells cultured in 20% FBS (FBS group) until passage 8. n = 3, ^*^ *P*<0.05 compared to FBS group. Data showed that CPD was significantly higher when hMDPCs were cultured in PRP rather than FBS.

Cumulative population doubling rates (CPD) were calculated to compare the effects of the different supplements (20% FBS or 20% PRP) on the long-term expansion of the hMDPCs. CPD was significantly higher when hMDPCs were cultured in PRP than in the FBS up to passage 8 (**Fig. 2**).

### PDGF Plays a Key Role in the Mitogenic/Proliferative Effects of PRP

**Figure 3:**
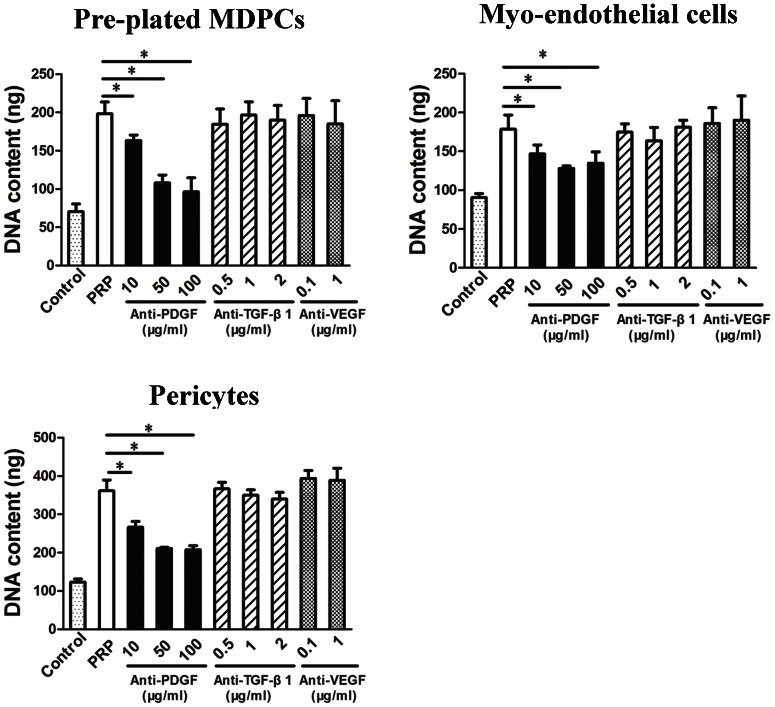
The role of growth factors in PRP on the proliferation of hMDPCs. Neutralizing antibodies against PDGF, TGF-β1, and VEGF were added to the media to determine the role that these growth factors played in the beneficial effect that PRP had on hMDPCs proliferation. n = 4, ^*^ *P*<0.05. Data showed that PDGF within the PRP played a role in stimulating the proliferation of the hMDPCs.

In order to test which growth factor(s) within PRP may be responsible for promoting the proliferation of hMDPCs; three major growth factor components of PRP were neutralized separately with the addition of neutralization antibodies. The effects that the neutralized PRPs had on the proliferation of hMDPCs were then compared to un-neutralized PRP. Anti-PDGF antibody inhibited the mitogenic/proliferative effects of PRP in all the hMDPC cultures in a dose-dependent manner (**Fig. 3** **, anti-PDGF)**. For example, in the pericyte cultures, the addition of 10 µg/ml anti-PDGF antibody resulted in a significant decrease in DNA content when compared to the PRP control. A higher concentration of anti-PDGF antibody (50 µg/ml) reduced proliferation of the cells by 56% compared with the un-neutralized PRP treated cells. Increasing the concentration of the anti-PDGF antibody to 100 µg/ml did not further decrease the proliferation of the hMDPCs when compared with the 50 µg/ml group, and did not completely block the proliferative activity of the cells to the levels obtained with serum-free media (Control). There were no significant changes in proliferation in any of the hMDPC groups treated with anti-TGF-β1 and anti-VEGF neutralizing antibodies (**Fig. 3** **, anti-TGF-β1 and anti-VEGF**); and no significant changes in proliferation were noticed when adding the isotype control Abs to the PRP supplemented hMDPC cultures (**Fig. S1**).

### PRP Expanded hMDPCs Maintained a Stem Cell Marker Expression Profile

To determine the influence that PRP supplementation had on the stem cell marker expression profile, we performed RT-PCR (**Tab. 2**) and flow cytometry analyses (**Tab. 3** **)** on long-term expanded hMDPCs. The hMDPCs were expanded initially for 5–8 passages in 20% FBS. The culture media was then either switched to media supplemented with 20% PRP or the cells continued to be cultured in 20% FBS. After three weeks of additional culturing, the expression profiles of the chosen markers outlined in Tables 2 and 3 were compared between the two groups and to the cells prior to their additional three weeks of expansion (control, 5–8 passages post-isolation).

**Table 2:** RT-PCR analysis.

mRNA	Pre-plated MDPCs			Myo-endothelial cells			Pericytes		
	Control	FBS	PRP	Control	FBS	PRP	Control	FBS	PRP
**CD105**	0.67±0.14	0.57±0.16	0.64±0.10	0.70±0.09	0.71±0.14	0.65±0.16	0.57±0.06	0.58±0.06	0.52±0.09
**CD73**	0.60±0.04	0.64±0.04	0.75±0.09* #	0.73±0.15	0.70±0.11	0.67±0.13	0.85±0.07	0.60±0.13	0.63±0.10
**CD90**	0.57±0.04	0.63±0.09	0.76±0.11* #	0.74±0.14	0.69±0.08	0.71±0.16	0.63±0.08	0.54±0.10	0.54±0.09
**CD44**	0.36±0.05	0.38±0.09	0.28±0.19	0.43±0.07	0.47±0.11	0.57±0.06* #	0.40±0.09	0.43±0.11	0.37±0.08
**PAX-2**	0.56±0.10	0.36±0.09	0.41±0.13	0.38±0.08	0.42±0.10	0.40±0.09	0.32±0.15	0.35±0.10	0.43±0.16
**BMPR-1A**	0.57±0.05	0.59±0.08	0.42±0.03* #	0.39±0.06	0.41±0.09	0.40±0.09	0.35±0.11	0.39±0.09	0.34±0.11
**BMPR-1B**	0.46±0.10	0.39±0.09	0.59±0.07* #	0.38±0.07	0.32±0.11	0.46±0.05* #	0.30±0.03	0.34±0.06	0.45±0.04* #
**BMPR-2**	0.24±0.06	0.28±0.09	0.34±0.03* #	0.30±0.05	0.28±0.03	0.37±0.05* #	0.21±0.09	0.28±011	0.24±0.08
**ALDH**	0.25±0.04	1.10±0.09*	0.99±0.08*	0.89±0.11	0.87±0.11	0.89±0.09	N/A	N/A	N/A
**Nanog**	0.37±0.09	N/A*	0.32±0.14#	0.28±0.03	0.31±0.11	0.43±0.08* #	0.13±0.06	0.19±0.06	0.19±0.03
**OCT-4**	1.69±0.17	1.25±0.12*	1.71±0.08#	1.43±0.31	0.13±0.02*	0.28±0.05* #	0.36±0.10	0.13±0.03*	0.33±0.06#
**SOX-2**	0.27±0.06	N/A*	0.39±0.07* #	0.34±0.05	0.32±0.11	0.49±0.03* #	0.20±0.12	N/A*	0.05±0.00#
**RUNX2**	0.99±0.16	1.01±0.25	0.90±0.22	1.25±0.09	1.13±0.19	1.20±0.12	1.03±0.06	0.98±0.11	1.12±0.21
**ALP**	0.15±0.04	0.19±0.04	0.18±0.08	0.19±0.09	0.17±0.10	0.21±0.11	0.17±0.06	0.15±0.08	0.13±0.05
**SOX9**	0.24±0.08	0.26±0.07	0.31±0.11	1.20±0.07	1.25±0.12	1.16±0.16	0.21±0.07	0.26±0.05	0.38±0.09* #
**Aggrecan**	0.09±0.01	0.19±0.06*	N/A* #	0.18±0.03	0.20±0.06	0.03±0.01* #	0.17±0.02	0.27±0.03*	0.13±0.05#
**CD56**	0.36±0.03	0.50±0.09*	0.38±0.04#	0.41±0.08	0.39±0.12	0.45±0.09	N/A	N/A	N/A
**Desmin**	0.26±0.02	0.32±0.04*	0.10±0.04* #	0.38±0.02	0.45±0.02*	0.34±0.03#	N/A	N/A	N/A

hMDPCs were expanded with FBS or PRP for 3 weeks. The controls were hMDPCs cultured at day0. Data shown are means ± SD.

n = 3,

* *P*<0.05 compare to control,

# *P*<0.05 compare to FBS group.

N/A means the expression level was not detectable.

**Table 3:** Flow cytometry analysis.

	Pre-plated MDPCs			Myo-endothelial cells			Pericytes		
	Control	FBS	PRP	Control	FBS	PRP	Control	FBS	PRP
**CD146**	99.25±0.21	98.95±0.30	90.43±5.35	99.63±0.05	99.78±0.10	96.40±0.75	98.65±1.17	99.10±0.22	94.30±1.73
**CD144**	77.34±14.41	91.27±6.55	75.24±24.05	94.30±5.75	97.45±2.90	98.58±0.71	2.10±2.40	1.51±0.22	0.33±0.23
**CD56**	86.60±21.28	75.58±10.82	71.57±2.31*	91.43±4.53	91.48±3.07	92.33±5.89	3.74±1.54	4.56±1.33	7.60±1.55
**CD34**	0.42±0.22	0.30±0.03	0.14±0.16	93.22±4.50	98.51±1.83	92.49±3.24	0.39±0.55	0.36±0.46	0.14±0.10
**CD45**	1.88±0.77	3.77±2.13	3.79±4.65	0.99±0.14	1.11±0.25	1.81±0.60	0.96±0.65	4.67±3.32	4.72±3.37
**CD105**	97.40±3.47	98.50±1.85	95.53±4.35	99.45±0.07	99.15±0.64	99.40±0.57	98.97±1.45	98.33±2.46	98.07±3.09
**CD44**	98.80±1.30	93.97±4.50	91.80±8.25	99.03±1.33	98.33±2.38	99.66±0.21	98.60±1.59	98.40±2.51	99.53±0.55
**CD90**	92.97±3.09	94.20±4.15	99.37±0.51	97.63±2.14	99.43±0.32	97.40±3.47	60.70±4.13	99.47±0.40*	99.60±0.30*
**HLA-DR**	2.52±0.66	1.70±0.52	3.44±3.14	0.35±0.05	0.74±0.14	0.43±0.05	0.35±0.07	1.86±0.31	0.49±0.11
**OCT4**	14.67±4.98	11.76±6.99	34.39±1.97* #	20.927±5.65	13.70±2.70	57.80±3.20* #	61.76±6.84	24.85±6.75*	52.34±6.94* #
**Nanog**	68.80±3.25	44.10±10.32*	52.85±6.44	30.80±4.67	32.15±9.55	57.95±6.43* #	4.51±5.79	40.45±5.73*	33.70±11.60*
**SOX2**	52.00±9.42	32.43±2.42*	68.50±2.86#	24.10±5.27	28.03±8.15	33.67±5.00*	41.37±3.86	63.53±5.07*	53.57±6.59* #

hMDPCs were expanded with FBS or PRP for 3 weeks. The controls were hMDPCs cultured at day0; FBS group was hMDPCs cultured in FBS supplemented media; PRP group was hMDPCs expanded in PRP supplemented media. The percentage of positively stained cells was quantified by flow cytometry. Data shown are mean ± SD. n = 3,

* *P*<0.05 compare to control,

# *P*<0.05 compare to FBS group.

Three groups of genes were tested via RT PCR including: 1) Markers for mesenchymal stem cells [37], [38]: CD105 (also known as endoglin), CD73 (also known as 5′ nucleotidase), CD90 (also known as Thy-1), CD44, PAX2, Bone morphogenetic protein receptors (BMPRs, including BMPR1A, 1B and 2), and aldehyde dehydrogenase (ALDH); 2) Transcription factors that play a key role in controlling stemness [37], [38]: Nanog, Oct4, and Sox-2; 3) Markers that are related to lineage specific differentiation: Runt-related transcription factor 2 (RUNX2), alkaline phosphatase (ALP), SOX9, aggrecan, CD56 (also known as neural cell adhesion molecule, NCAM), and desmin.

After expansion in the PRP supplemented media, the mRNA expression levels of the preplated hMDSCs were significantly elevated in 6 of 9 of the mesenchymal stem cell (MSC) marker genes analyzed compared to both the low passage control cells and the FBS long term expanded cells (**Tab. 2**). MSC marker gene mRNA levels in the myo-endothelial cells were also significantly higher in 3 of 9 genes analyzed compared to both the low passage control cells and the FBS long term expanded cells (**Tab. 2**). Only 1 of 9 of the genes was significantly elevated in the PRP cultured pericytes compared to both the low passage control cells and the FBS long term expanded cells **(** **Tab. 2** **)**.

Nanog, OCT4, and SOX2 mRNA levels were significantly elevated in the PRP supplemented preplate hMDPCs, myo-endothelial cells, and pericytes compared to the FBS supplemented cultures in 2 of 3 (Nanog and Sox-2), 3 of 3, and 2 of 3 (Oct4 and Sox-2) of the genes analyzed, respectively (**Tab. 2**). The PRP supplemented cells had significantly elevated gene expression levels in 1 of 3 (Sox-2), 3 of 3, and 0 of 3 (preplate hMDPCs, myo-endothelial and pericytes, respectively) genes, compared to the early passage control cells **(** **Tab. 2** **).**

The PRP supplemented populations of cells showed no significant increase in lineage specific mRNA expressions compared to the early passage control cells, except for the pericytes which exhibited an increase in Sox-9 expression. The PRP supplemented cultures, however, did show a significant decrease in the expression of certain markers compared to both the early passage control cells and the long term FBS supplemented cultures (**Tab. 2**). The real-time RT-PCR data on one population of each hMDPCs showed similar result (**Tab. S1**).

The marker profiles of the hMDPCs after PRP or FBS expansion were further analyzed by flow cytometry (**Tab. 3**). The cell surface markers used to isolate myoendothelial cells and pericytes (CD146, CD144, CD56, CD34, and CD45) demonstrated no significant changes in positivity after expansion in either FBS or PRP supplemented cultures (**Tab. 3**). The pre-plated hMDPCs showed a heterogeneous phenotype and highly expressed CD146 (99.25%±0.21), CD144 (77.34%±14.41), CD56 (86.60%±21.28), but not CD34 (0.42%±0.22); however, no significant change to this marker profile was found after expansion in either the FBS or PRP supplemented media (**Tab. 3**).

Mesenchymal stem cell surface markers, CD105, CD44, and CD90, were highly expressed by all three hMDPC populations in the early passage control cells as well as by all the expanded populations supplemented with either PRP or FBS (**Tab. 3**), and no significant changes in positivity were found for any of these markers in any of the hMDPC populations. Interestingly, the mean fluorescent intensity (MFI) of CD105 and CD90 were significantly higher in all the PRP expanded hMDPCs compared to the control and FBS expanded cells. For example, in the pre-plated MDPCs, the MFI of CD90 and CD105 was significantly higher in the PRP group (CD90: Control, 29247.25±5196.54; FBS, 41329.00±4032.05; PRP, 86618.00±7765.70; CD105: control, 4258.00±506.17; FBS, 5729.60±592.80; PRP, 9179.40±625.98).The expression of Nanog, Oct4, and Sox-2 were also detected via flow cytometry in all the early passage control populations (**Tab. 3** **control**); after expansion, all three of the PRP supplemented hMDPC populations yielded significantly higher expression in at least 1 of 3 markers compared to the FBS supplemented and low passage control populations **(** **Tab. 3** **)**. For example, Oct4 was expressed significantly more in all three populations of the PRP supplemented cells, Nanog was expressed significantly more by the myo-endothelial PRP supplemented cells than the early passage control and the FBS supplemented long term cultures, as was Sox-2 by the pericyte population (**Tab. 3**).

### PRP Expanded hMDPCs Maintained their *in vitro* Differentiation Capabilities

The differentiation abilities of all three hMDPC populations were compared after three weeks in either the PRP (20%) or the FBS (20%) supplemented media.

**Figure 4:**
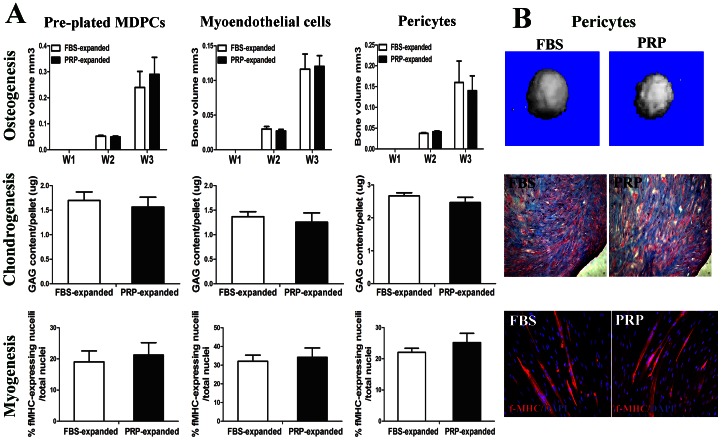
Differentiation abilities of hMDPCs were maintained after expansion with PRP. hMDPCs expanded either with PRP or FBS were tested for their multi-lineage differentiation abilities. **A.** For osteogenesis, data showed the mineralized bone volume of the pellets at different time points; for chondrogenesis, data showed GAG content of pellets; for myogenesis, data showed the percentage of f-MHC expressing nuclei per total nuclei. **B.** representative data of pericytes. For osteogenesis, pictures of 3D mineralization within the pellets; for chondrogenesis, alcian blue staining of the pellets; for myogenesis, immuno-staining of f-MHC (red) and nuclei (blue). Cells expanded in both culture conditions consistently differentiated into osteogenic, chondrogenic, and myogenic lineages. There were no significant differences in regard to the multi-lineage differentiation ability between the PRP-expanded and FBS-expanded hMDPCs (n = 3).

For osteogenesis, micro-CT scanning revealed an increase in bone volume in all the hMDPCs cell pellets in both groups in a time dependent manner. No significant differences were observed between the PRP and FBS supplemented groups (**Fig. 4** **Osteogenesis)**.

For chondrogenesis, one population of myo-endothelial cells and pericytes from one donor did not positively respond to chondrogenic induction. All the other populations of hMDPCs formed cartilage-like pellets after four weeks of induction. All the pellets contained a significant amount of glycosaminoglycan (GAG), and were positive for alcian blue staining (**Fig. 4** **Chondrogenesis)**; however, no significant differences were observed between the PRP and FBS supplemented groups.

For myogenesis, human fast myosin heavy chain (f-MHC) positive myotubes were formed in both groups on day ten. No significant differences were observed in the percentages of f-MHC-expressing nuclei/total nuclei when compared between the two groups (**Fig. 4** **Myogenesis)**.

### PRP-expanded hMDPCs Maintained their *in vivo* Myogenic Potential

**Figure 5:**
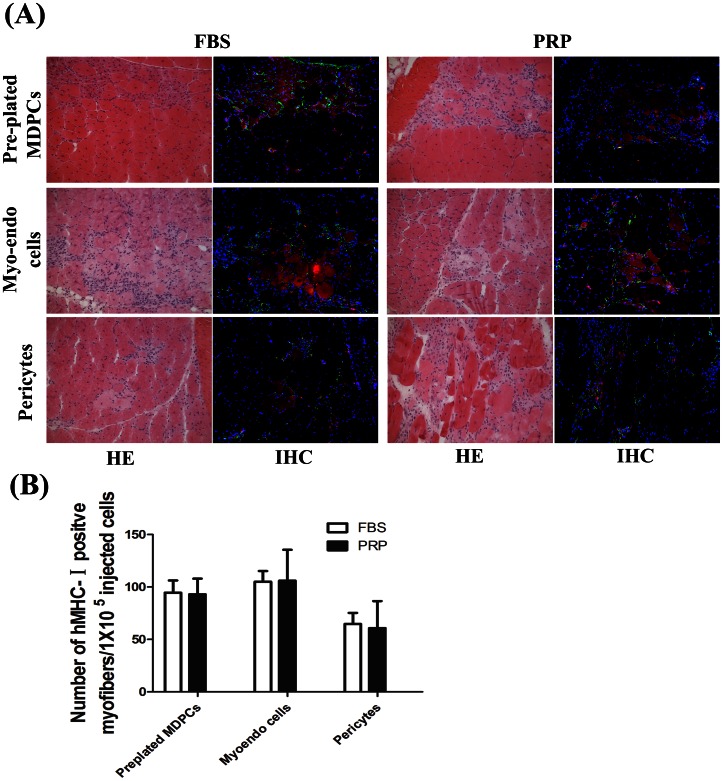
*In vivo* myogenic potential of PRP-expanded hMDPCs. PRP- and FBS-expanded hMDPCs were injected into mdx-SCID gastrocnemius muscles damaged with cardiotoxin. Cryo-sections were prepared 4 weeks after cell injection. **A.** HE staining and immuno-fluorescence staining of human major histocompatibility complex (MHC) class-I (red), human mitochondria (green), and DAPI (blue) were performed on the slides that contained the injection sites. Human MHC-I-positive and human mitochondria-positive myofibers were detected in the injured area. **B.** Quantification of human MHC-I-positive fibers per 1×10^5^ injected cells. Data showed that all populations of hMDPCs that were expanded in PRP supplemented media retained their ability to regenerate myofibers, and no significant differences were found between FBS and PRP expanded cells (n = 4).

All three populations of PRP-expanded and FBS-expanded hMDPCs were injected into the gastrocnemius muscles of mdx-SCID mice damaged with cardiotoxin. The mean cumulative population doublings rate (CPD) at the time of transplantation of the PRP-expanded cells were 29.30 (pre-plated hMDPCs), 33.09 (myo-endothelial cells), and 31.57 (pericytes); and the CPD of FBS-expanded cells were 18.73 (pre-plated hMDPCs), 21.84 (myo-endothelial cells), and 17.78 (pericytes). Four weeks after cell injection, human Major histocompatibility complex (MHC) class I (red) and human mitochondria positive (green) myofibers could be detected in the injured muscles (**Fig. 5** **A, Fig. S2** shows secondary antibody only, control). Human MHC-class-I was mainly expressed on the membrane and in the sarcoplasm of the regenerated muscle fibers [39]; human mitochondria could be detected in the injured area, both in the periphery of the human MHC-class-II positive muscle fibers and around the transplanted area [40]. All populations of hMDPCs that were expanded in PRP supplemented media retained their ability to regenerate myofibers upon extended *in vitro* culture. The number of human MHC-class-I positive myofibers was quantified and no significant differences were found between the PRP and FBS expanded hMDPC populations **(** **Fig. 5** **B)**.

## Discussion

PRP is heterogeneous by nature due to the numerous variations that exist in PRP preparation protocols including: 1) the starting number of platelets, 2) the use of anticoagulants, 3) the inclusion of leukocytes, and 4) the use of activators which can lead to different biological effects imparted by the PRP [41], [42], [43], [44], [45]. The PRP used in this study was leukocyte and platelet-rich plasma (L-PRP), and is classified as Type 2A according to Mishra, A., *et al*.’s classification system [46]. PRP from six donors were pooled and the concentration of the platelets in the pooled PRP was adjusted to 2×10^6^ platelets per microliter (around 10 times above the baseline found in blood). The level of three primary growth factors in the PRP (PDGF-AB, TGF-β1, and VEGF) [23], [43] was determined, and our results demonstrated that the pooled PRP contained significant amounts of PDGF-AB and TGF-β1, and a fair amount of VEGF, which is consistent with previous reports from other groups [27], [47]. Although autologously derived PRP is preferable to avoid possible immune cross reactions, intrinsic variations [43] such as altered platelet quantity and quality caused by sex, age, and pre-existing patient conditions, [48], [49], [50] make it difficult to control the quality of individual PRPs. Obtaining large amounts of autologous PRP for expansion of stem cells *in vitro* could also be problematic, as a result, allogeneic PRP drew our attention and allogeneic platelet transfusion is well established in the clinical setting [51], [52]. Screened and tested allogeneic platelet concentrates are available from blood banks in bulk, and their quantity and quality are tested and controlled before use to confirm their safety and effectiveness; however, a lack of well-accepted quality control criteria for PRP exists, largely because of the unknown mechanism of action of PRP and the complexity of its components [23], [53]. In this study, we normalized the concentration of platelets, and the concentrations of major growth factors in the PRP were tested; however, more specific quality control criteria of PRP for specific applications require further investigation.

Prior *in vitro* studies consistently demonstrated that PRP enhanced the proliferation of a variety of human cell types including MSCs derived from bone marrow [25], [29], [54], [55], [56], [57], [58] and adipose tissue [31]. For the first time, both short-term and long-term proliferation data revealed that PRP also had a potent effect on the proliferation of hMDPCs, both in the presence and absence of FBS. The PRP expanded hMDPCs maintained their stem cell marker expression profile more effectively than the FBS expanded cells. The PRP-expanded cells also maintained their multi-lineage differentiation capacity, which was confirmed by the cells’ ability to differentiate into osteogenic and chondrogenic lineages *in vitro* and a myogenic lineage both *in vitro* and *in vivo*. Taken together, our data suggest that PRP could be used as a supplement for the expansion of hMDPCs in place of FBS. To our knowledge, this is the first study to demonstrate the efficacy and safety of PRP on the *ex-vivo* expansion of hMDPCs. The use of PRP for hMDPC expansion has the potential to reduce the cost of cell culture and increase the safety of this cell-based protocol due to the fact that both PRP and hMDPCs can be collected autologously or, under well-controlled conditions, allogeneically.

It is well established that a number of markers associated with the stemness of somatic stem cells are expressed simultaneously by uncommitted MSCs, and their expression levels decrease once the stem cells commit to a specific lineage [37], [38]. It is critical to keep the stable expression of those markers during their initial *ex-vivo* expansion for later transplantation. In the current study, MSC markers and stem cell transcription factors were chosen to examine the effect of PRP-supplementation on the stem cell phenotype of the hMDPCs. Among these markers, Nanog, Oct4, and Sox-2 (markers for embryonic stem cells) were used to demonstrate potential pluripotency [59], [60], [61], [62], [63]. Recent studies demonstrated that these transcription factors are also expressed by adult stem cells [64], [65], [66], [67], and we found that these transcription factors and mesenchymal stem cell markers were indeed expressed by the hMDPCs. A more stable and sustained expression of these markers was observed in cells that were expanded in the PRP-supplemented media when compared with cells that were expanded in the FBS-supplemented media. One of the first steps in the commitment of MSCs to differentiate into tissue specific regenerating cells is a shift in the balance of these stem cell markers in favor of lineage specific markers. In the current study we observed a reduction in the expression of lineage specific markers by the PRP-expanded cells, which further supported our hypothesis. Moreover, PRP was also shown to have a strong mitogenic/proliferative effect on the hMDPCs, which is additional evidence that PRP maintained the cells in a less differentiated state. Since proliferation and differentiation have been shown to be mutually exclusive in MSCs [68], failure to exit the cell cycle could be responsible for preventing the hMDPCs from entering into a differentiation phase.

In this study, we investigated three major growth factors (PDGF, TGF-β1, and VEGF) [23], [43] that could contribute to the mitogenic/proliferative effects that PRP had on the hMDPCs. Our data showed that neutralization of PDGF partially inhibited the mitogenic/proliferative effects that PRP had on the hMDPCs while neutralization of TGF-β1 and VEGF had little effect. These findings suggest that the mitogenic effect that PRP had on the hMDPCs is, at least in part, mediated by PDGF. It is well known that there are more than three hundred growth factors within PRP and that the function of PRP is multifactorial. In addition to PDGF [69], [70], [71], TGF-β1 [72], [73], and VEGF [74], many other platelet-derived factors have been studied individually and have been shown to strongly influence cellular proliferation and differentiation; for example, insulin-like growth factor (IGF) [75], [76], [77] and epidermal growth factor (EGF) [78], [79]. PRP contains high concentrations of all of these factors which probably work synergistically. Further comprehensive studies are required to fully understand the mechanisms behind the actions of PRP.

This study demonstrated that PRP promoted the proliferation of hMDPCs and preserved their multi-linage differentiation capacity during *ex-vivo* expansion. PDGF appears to be a key growth factor within PRP that contributes to the mitogenic/proliferative effects that PRP imparts on hMDPCs. We therefore conclude that PRP could be used as an excellent supplement for the *ex-vivo* expansion of hMDPCs; however, PRP’s major limitation is its heterogeneity which requires the need for quality testing prior to use.

## Supporting Information

Figure S1 **Negative controls of neutralization assay.** Low endotoxin isotype control Abs were added (goat IgG, 100 µg/ml; rabbit IgG, 2 µg/ml; mouse IgG, 1 µg/ml) to the 10% PRP supplemented media as controls. No significant changes in proliferation were noticed when adding the isotype control Abs to the PRP supplemented hMDPC cultures compare to the PRP groups (n = 4).
(TIF)

Figure S2 **Second antibody alone controls for immunostaining of muscle sections.** No fluorescent signals were detected. DAPI (blue).
(TIF)

Table S1 **Real-time RT-PCR analysis.** Cellular RNA of hMDPCs was extracted using an RNeasy Mini Kit (Qiagen). Aliquots of 1 µg total RNA were hybridized with random primers and converted into cDNA using a SuperScript First-Stand Synthesis System (Invitrogen). Real time PCR was performed on an iCycler iQ5 PCR machine (BioRad) using SYBR Green Master mix (Thermo Scientific). The gene-specific primer sets were used at a final concentration of 0.3 µM. All real time PCR assays were performed in triplicates. Gene expression was calculated using the relative standard curve method. Expression of the specific markers were normalized to β-actin and then scaled according to the control sample. This value was set to 1. Values are average of the triplicates.
(DOCX)
