# Supplementary figures and images for: Platelet-Rich Plasma Promotes the Proliferation of Human Muscle Derived Progenitor Cells and Maintains Their Stemness
Source: PLoS One. 2013 Jun 7;8(6):e64923. doi: 10.1371/journal.pone.0064923 (PMC3676442; doi:10.1371/journal.pone.0064923)

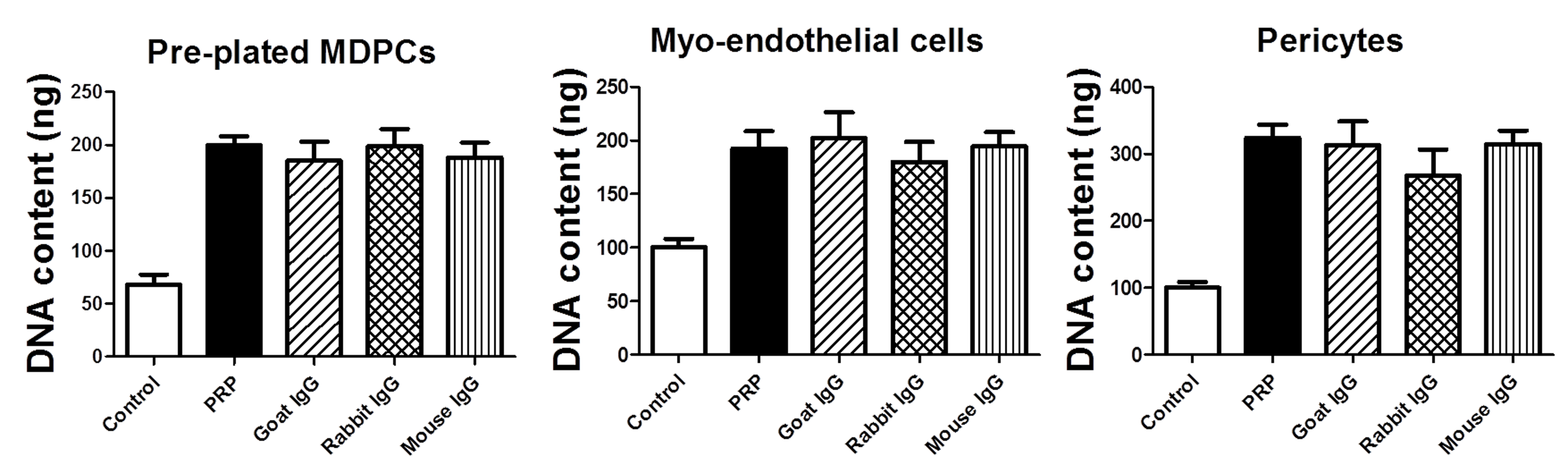

Supplement: Figure S1 — Negative controls of neutralization assay. Low endotoxin isotype control Abs were added (goat IgG, 100 µg/ml; rabbit IgG, 2 µg/ml; mouse IgG, 1 µg/ml) to the 10% PRP supplemented media as controls. No significant changes in proliferation were noticed when adding the isotype control Abs to the PRP supplemented hMDPC cultures compare to the PRP groups (n = 4). (TIF) [file pone.0064923.s001.tif]

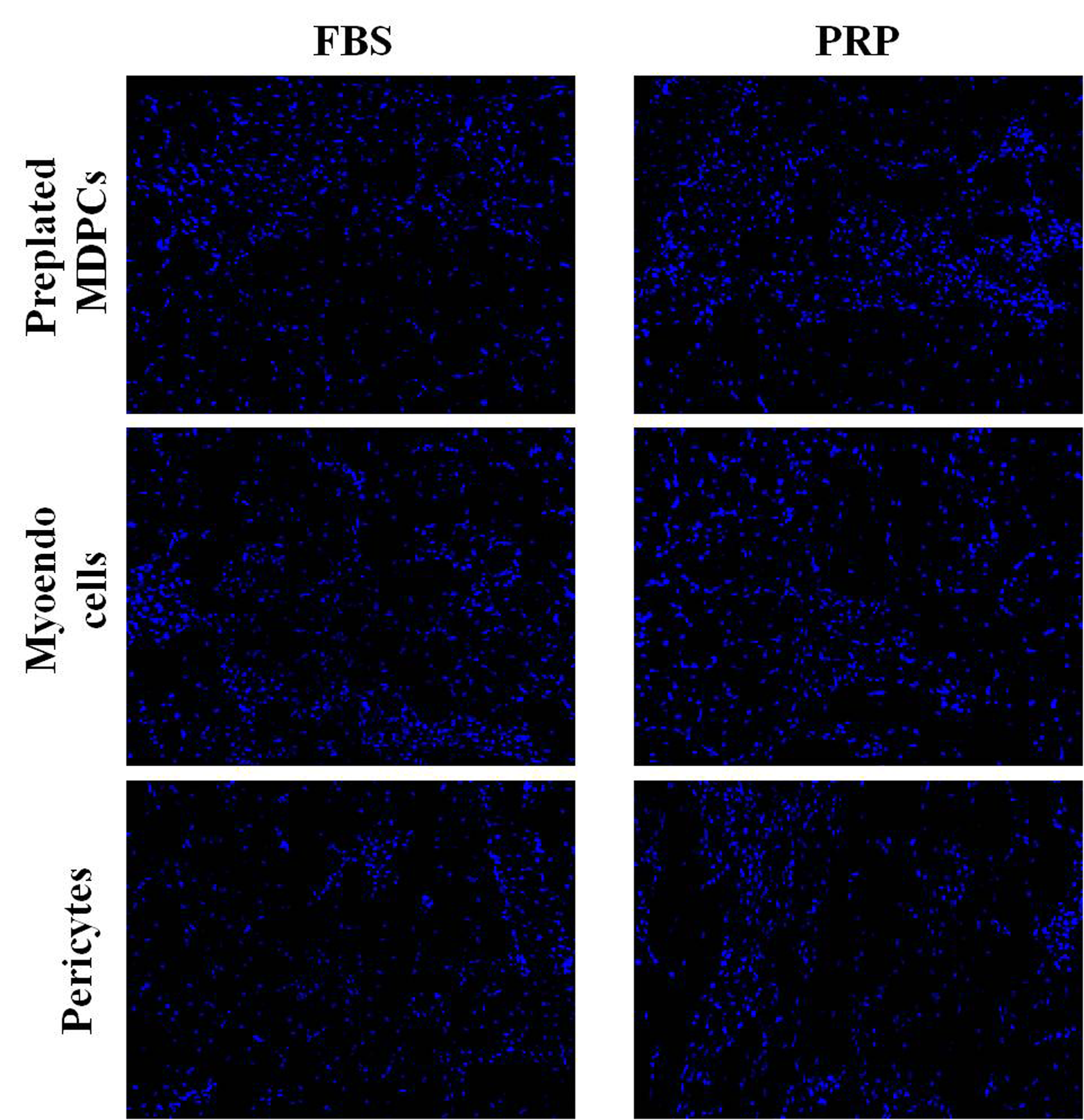

Supplement: Figure S2 — Second antibody alone controls for immunostaining of muscle sections. No fluorescent signals were detected. DAPI (blue). (TIF) [file pone.0064923.s002.tif]
