# Supplementary material for: Platelet-Rich Plasma Promotes the Proliferation of Human Muscle Derived Progenitor Cells and Maintains Their Stemness
Source: PLoS One. 2013 Jun 7;8(6):e64923. doi: 10.1371/journal.pone.0064923 (PMC3676442; doi:10.1371/journal.pone.0064923)
Supplement: Table S1 — Real-time RT-PCR analysis. Cellular RNA of hMDPCs was extracted using an RNeasy Mini Kit (Qiagen). Aliquots of 1 µg total RNA were hybridized with random primers and converted into cDNA using a SuperScript First-Stand Synthesis System (Invitrogen). Real time PCR was performed on an iCycler iQ5 PCR machine (BioRad) using SYBR Green Master mix (Thermo Scientific). The gene-specific primer sets were used at a final concentration of 0.3 µM. All real time PCR assays were performed in triplicates. Gene expression was calculated using the relative standard curve method. Expression of the specific markers were normalized to β-actin and then scaled according to the control sample. This value was set to 1. Values are average of the triplicates. (DOCX) [file pone.0064923.s003.docx]

**Table S1**

| mRNA | Pre-plated MDPCs | | | Myo-endothelial cells | | | Pericytes | | |
| --- | --- | --- | --- | --- | --- | --- | --- | --- | --- |
|  | **Control** | **FBS** | **PRP** | **Control** | **FBS** | **PRP** | **Control** | **FBS** | **PRP** |
| CD105 | 1 | 1.34 | 1.24 | 1 | 1.47 | 1.56 | 1 | 1.04 | 2.16 |
| CD73 | 1 | 1.23 | 1.81 | 1 | 0.83 | 1.72 | 1 | 0.83 | 1.10 |
| CD90 | 1 | 1.22 | 2.89 | 1 | 1.26 | 4.12 | 1 | 1.44 | 1.22 |
| CD44 | 1 | 0.87 | 0.62 | 1 | 0.50 | 2.05 | 1 | 0.86 | 0.91 |
| PAX-2 | 1 | 1.19 | 0.92 | 1 | 1.45 | 2.64 | 1 | 0.47 | 1.57 |
| BMPR-1A | 1 | 1.46 | 0.40 | 1 | 1.17 | 1.42 | 1 | 1.09 | 1.08 |
| BMPR-1B | 1 | 0.52 | 2.29 | 1 | 1.95 | 6.28 | 1 | 0.32 | 2.69 |
| BMPR-2 | 1 | 1.15 | 2.65 | 1 | 0.54 | 2.31 | 1 | 0.87 | 0.88 |
| ALDH | 1 | 1.32 | 2.13 | 1 | 0.66 | 0.80 | 1 | 0.29 | 0.63 |
| Nanog | 1 | 0.08 | 0.91 | 1 | 1.03 | 3.39 | 1 | 1.21 | 2.50 |
| OCT-4 | 1 | 0.25 | 1.44 | 1 | 0.98 | 1.64 | 1 | 0.66 | 2.30 |
| SOX-2 | 1 | 0.17 | 1.84 | 1 | 1.08 | 2.21 | 1 | 0.53 | 0.98 |
| RUNX2 | 1 | 2.15 | 0.54 | 1 | 1.35 | 1.02 | 1 | 0.61 | 0.76 |
| ALP | 1 | 0.71 | 0.70 | 1 | 1.39 | 1.45 | 1 | 1.50 | 1.55 |
| SOX9 | 1 | 1.64 | 1.51 | 1 | 0.78 | 0.97 | 1 | 0.83 | 2.18 |
| Aggrecan | 1 | 13.10 | 0.06 | 1 | 2.05 | 0.58 | 1 | 2.68 | 0.30 |
| Desmin | 1 | 0.92 | 0 | 1 | 2.01 | 0.90 | 1 | 0.82 | 0.13 |
